# Genome-wide identification and functional characterization of *LBD* transcription factors uncover their stress adaptation mechanisms in tobacco and eggplant

**DOI:** 10.3389/fpls.2026.1958148

**Published:** 2026-09-18

**Authors:** Tianyi Wu, Binghui Zhang, Qitang Guo, Yani Li, Chengjie Li, Qian He, Dehan Chen, Gang Gu, Anqi Chen, Zongfeng Yang, Wenqing Li, Xiaofang Xie

**Affiliations:** 1 College of Life Sciences, Fujian Agriculture & Forestry University, Fuzhou, China; 2 Institute of Tobacco Science, Fujian Provincial Tobacco Company, Fuzhou, China; 3 Sanming Tobacco Company, Sanming, China; 4 Tobacco-Rice Synergistic Development Industrial Technology Engineering Center, State Tobacco Monopoly Administration (China National Tobacco Corporation), Fuzhou, China; 5 Fujian Key Laboratory of Crop Breeding by Design, Fujian Agriculture & Forestry University, Fuzhou, China

**Keywords:** eggplant, evolutionary analysis, functional characterization, LBD, stress, tobacco, VIGS

## Abstract

The Lateral Organ Boundaries Domain protein family comprises a group of plant-specific transcription factors involved in diverse developmental and stress-response processes. We identified 106 *NtLBD* genes in tobacco (*Nicotiana tabacum* L.) and 50 *SmLBD* genes in eggplant (*Solanum melongena* L.), and classified them into Classes I and II. Duplication analysis identified 26 segmental and 3 tandem duplicated gene pairs, while interspecies synteny analysis revealed 17 tobacco–*Arabidopsis thaliana* and 31 eggplant–*Arabidopsis thaliana* syntenic pairs. Expression profiling showed broad tissue expression, with relatively high transcript abundance in roots and stems, and selected LBD proteins were localized to the nucleus. VIGS constructs targeting *NtLBD4/NtLBD31* in tobacco and *SmLBD19/SmLBD48* in eggplant were used for preliminary functional assessment. In tobacco, these treatments were associated with reduced expression of ABA-responsive genes, less visible injury under low-temperature stress, and increased susceptibility to bacterial wilt. In eggplant, they were associated with increased expression of ABA biosynthesis and signaling related genes, reduced visible cold injury, and enhanced bacterial wilt resistance. Expression changes in related LBD members further suggested possible functional redundancy or compensatory regulation. These findings indicate that selected *LBD* genes and their associated regulatory networks participate in ABA responses, low-temperature adaptation, and pathogen defense in tobacco and eggplant, and they further underscore the potential utility of these genes as targets for improving stress tolerance in *Solanaceous* species, although additional experimental confirmation is warranted.

## 1 Introduction

The Lateral Organ Boundaries Domain (LBD) proteins constitute a plant-specific family of transcription factors, characterized by a conserved N-terminal Lateral Organ Boundary (LOB) domain (Yu et al., 2020). This family was first identified in *Arabidopsis thaliana* when “Lateral Organ Boundaries (*LOB/AtASL4*)”, a gene regulating adaxial side development in lateral organs, was characterized via enhancer trapping (Shuai et al., 2002). Shortly after, *ASYMMETRIC LEAVES 2 (AS2)* was found to share molecular and genetic features with *LOB*, formally establishing the *LBD* family (Iwakawa et al., 2002; Rong et al., 2024). The conserved LOB domain spans approximately 100 amino acids and consists of three conserved motifs: a CX_2_CX_6_CX_3_C zinc finger motif essential for DNA binding, a Gly-Ala-Ser-rich (GAS) region that modulates DNA-binding affinity, and a leucine-zipper-like coiled-coil motif (LX_6_LX_3_LX_6_L) responsible for protein dimerization (Wu et al., 2024; Liu et al., 2024). Based on the integrity of this leucine-zipper motif, LBD proteins are divided into two major classes: Class I, which retains a complete motif and is primarily involved in developmental processes such as leaf, root, flower development, bud initiation, plant regeneration, and secondary growth (Wei et al., 2022; Liu et al., 2023; Ye et al., 2021); and Class II, which contains a degenerate leucine-zipper and is mainly associated with abiotic stress responses, nitrogen metabolism, and lateral organ development (Graeff et al., 2016; Xiong et al., 2022).

Functional studies reveal both conserved and divergent roles among *LBD* members. In rice, *OsLBD37* and *OsLBD38* delay flowering by down-regulating *Ehd1*, leading to increased yield (Li et al., 2017). Similarly, *MdLBD11* overexpression delays flowering in apple (Wang et al., 2013). In wheat, however, overexpression of *TaLBD16-4D* promotes early maturity (Wang et al., 2022), highlighting species-specific regulatory roles. Furthermore, *LBD* members can engage in coordinated feedback regulation. For example, *PagLBD1* and *PagLBD3* fine-tune secondary growth through negative feedback in poplar (Han et al., 2021). In maize, *ZmLBD2* enhances abiotic stress tolerance, as evidenced by improved germination, root growth, water retention, chlorophyll and proline content, and antioxidant enzyme activity in overexpressing *Arabidopsis thaliana* lines (Jiao et al., 2022).

Tobacco and eggplant are economically important members of the *Solanaceae* family, with tobacco serving as both a widely used model system and a major commercial crop, and eggplant representing a globally cultivated vegetable of considerable nutritional and economic value (Pebriana et al., 2018; Chen et al., 2017; Tong et al., 2025). Despite their close taxonomic relationship, the two species differ markedly in their biological characteristics and agronomic uses, providing a complementary basis for comparative analysis of the conservation and diversification of the *LBD* gene family within *Solanaceae*. In addition, the well-established molecular and genetic resources available for tobacco, together with its suitability for virus-induced gene silencing (VIGS), facilitate functional characterization of candidate genes, whereas the inclusion of eggplant extends the analysis to an important *solanaceous* vegetable crop. Although the *LBD* family has been extensively characterized in model and crop species such as *Arabidopsis thaliana* (Shuai et al., 2002; Matsumura et al., 2009), *Vitis vinifera* (Cao et al., 2016), *Hordeum vulgare* L (Guo et al., 2016), and *Zanthoxylum armatum* DC (Deng et al., 2025), comprehensive characterization of this gene family in tobacco and eggplant remains limited, and the biological functions of their *LBD* members are still poorly understood. Both crops are frequently exposed to abiotic and biotic stresses that adversely affect plant growth, productivity, and crop performance. Therefore, elucidating the roles of *LBD* genes in response to hormone, low-temperature, and pathogen responsive processes is critical for understanding stress regulatory mechanisms and for identifying candidate genes to enhance stress resilience. In contrast to studies primarily focused on gene family identification and expression profiling, the present study integrates comparative genome-wide analysis with functional validation in two economically important *Solanaceae* species. We first performed genome-wide identification and characterization of the *LBD* gene family and analyzed their tissue specific expression patterns and stress responsive profiles using transcriptome data. Four candidate genes (*NtLBD4, NtLBD31* in tobacco, *SmLBD19, SmLBD48* in eggplant) were then selected for loss-of-function assays via VIGS, and their roles were evaluated under abscisic acid (ABA) stress, low-temperature stress, and bacterial pathogen *Ralstonia solanacearum* (*Ras*) infection. Our findings provide a genetic foundation for future functional studies of the *LBD* family and trait improvement in these two *Solanaceous* crops.

## 2 Materials and methods

### 2.1 Identification and characterization of *LBDs* in tobacco and eggplant genome

To identify *LBD* gene family members at the genome-wide level in tobacco and eggplant, 43 previously reported *Arabidopsis thaliana* LBD protein sequences (*AtLBDs*) (Mangeon et al., 2012) were retrieved from the TAIR database and used as query sequences. Genome assemblies, predicted protein sequences, and corresponding GFF3 annotation files for the two species were obtained from the following sources: for tobacco, data were downloaded from the Sol Genomics Network (SGN; https://solgenomics.net/) based on the Edwards 2017 (Edwards et al., 2017) genome assembly; for eggplant, data were retrieved from the Eggplant Genome Database (http://47.92.172.28:12068) based on the Eggplant-HQ v1.0 assembly (Wei et al., 2020). Local BLAST searches were performed using the *AtLBD* sequences as queries against the nucleotide and protein datasets of tobacco and eggplant, respectively. BLASTP searches were conducted in TBtools-II v2.4 (Chen et al., 2023) with default parameters and an E-value cutoff of 1e^-10^. Sequences exhibiting a minimum sequence similarity of 30% were selected as candidate homologs. All non-redundant candidates were then examined using the Pfam database (https://www.ebi.ac.uk/interpro/search/, PF03195) and SMART (http://smart.embl-heidelberg.de/) to verify the presence of the conserved LOB domain, and only proteins containing an intact LOB domain were used for subsequent analyses. Finally, the basic physicochemical properties of the identified proteins, including amino acid length, molecular weight (MW), and theoretical isoelectric point (pI), were calculated using the ExPASy ProtParam tool.

### 2.2 Analysis of *cis*-acting elements in *LBD* gene promoters

The upstream 2000 bp sequences from the start codons of *NtLBD* and *SmLBD* genes were extracted using the Gtf/Gff3 Sequence Extracter tool in TBtools-II v2.4 (Chen et al., 2023) for promoter analysis. These sequences were then submitted to the PlantCARE online website (https://bioinformatics.psb.ugent.be/webtools/plantcare/html/) (Lescot et al., 2002) for predicting *cis*-acting elements.

### 2.3 Chromosomal locations and gene duplication analysis of *LBD* genes

The chromosomal positions of the identified *LBD* genes in tobacco and eggplant were precisely mapped using TBtools-II v2.4 (Chen et al., 2023). This information was used to annotate the specific genomic loci harboring the *LBD* genes. To examine potential gene duplication events, collinear relationships among *LBD* homologs were analyzed and visualized using the Circos function in TBtools-II v2.4 (Chen et al., 2023).

### 2.4 Phylogenetic analysis of LBD proteins

The candidate LBD protein sequences were aligned using ClustalX software. Based on this alignment, a phylogenetic tree for the *LBD* family was constructed in MEGA 11 (Kumar et al., 2016) using the Neighbor-Joining (NJ) algorithm, with branch reliability evaluated using 1000 bootstrap replicates.

### 2.5 Expression analysis of *LBD* genes in different tissues

To investigate the tissue-specific expression of *LBD* genes, the RNA-Seq data for *NtLBD* genes in different tissues (GSE233199; Mo et al., 2023) were retrieved from NCBI (https://www.ncbi.nlm.nih.gov/geo/). Similarly, the RNA-Seq data for *SmLBD* genes in various tissues (PRJNA839598; Liu et al., 2023) were also obtained. Expression heatmaps for *NtLBD* and *SmLBD* genes were generated using the HeatMap function in TBtools-II v2.4 (Chen et al., 2023).

### 2.6 Subcellular localization analysis of *LBDs*

Transient overexpression of *NtLBD4*, *NtLBD31*, *SmLBD19*, and *SmLBD48* was performed in *Nicotiana benthamiana* leaves via *Agrobacterium*-mediated transformation. The coding sequences of each gene were amplified with adapter-containing primers (**Supplementary Table S1**), designed using Primer Premier 5. PCR products were gel-purified and ligated into *SalI/KpnI*-digested pSuper1300-GFP to generate C-terminal GFP fusions. The resulting constructs were transformed into *E. coli* DH5α, and positive clones were selected on Kan^+^ LB plates. Plasmids were then transferred into *Agrobacterium tumefaciens* GV3101 (Wang et al., 2012) for leaf infiltration. After 48 h of incubation at 25 °C in darkness, GFP fluorescence in leaf cells was visualized and recorded using laser confocal microscopy.

### 2.7 VIGS vector construction and *Agrobacterium* transformation

Tobacco cultivar K326 and eggplant cultivar Yanzhiqie (YZQ; accession ID: V06B0149) were used in this study and were grown from seeds provided by the Fujian Key Laboratory of Crop Breeding by Design at Fujian Agriculture and Forestry University, in a greenhouse also provided by the same laboratory. The tobacco plants were grown in a greenhouse under the following conditions: a temperature of 28 °C a 16 h light/8 h dark photoperiod, and a light intensity of 200 × 100 Lux. The VIGS assay was performed according to an established protocol (Liu et al., 2002). A qTRV2 vector carrying the GFP gene sequence was included as an indicator of system efficiency, where GFP fluorescence signified successful expression. This system was then used to functionally characterize the selected *LBD* genes (*NtLBD4* and *NtLBD31* in tobacco, and *SmLBD19* and *SmLBD48* in eggplant) under biotic and abiotic stress conditions. The optimal gene-silencing fragment for each target was selected using the SGN VIGS Tool (https://vigs.solgenomics.net/), and the corresponding primers were designed with Primer Premier 5 (**Supplementary Table S2**). The fragment with added adaptor was amplified by nested PCR, gel-purified, and ligated into *BamHI/EcoRI*-digested pTRV2 to generate the pTRV2-*LBD* silencing construct. Following transformation into *E. coli* DH5α, positive clones were verified by PCR and restriction digestion. The confirmed plasmid was then transformed into *Agrobacterium tumefaciens* GV3101. For infiltration, cultures of pTRV2-*LBD*, the empty pTRV2 vector, and pTRV1 were grown in LB medium supplemented with Kan^+^ and Rif to an OD_600_ of 0.6-0.8. The cells were harvested, resuspended in infiltration buffer, and mixed at a 1:1 ratio (pTRV2:pTRV1) before being incubated in the dark for 4 h. The resulting suspensions were infiltrated into leaves of the common tobacco cultivar K326. Infiltrated plants were kept in darkness at 25 °C for 48 h, then transferred to a light cycle for 10–15 days to allow the development of GFP expression and visible phenotypes (Li et al., 2024a). Leaves were then exposed to a handheld UV flashlight (365 nm) to observe GFP expression, and the appearance of green fluorescence served as a visual indicator of viral infection. To detect the presence of the viral vector in the infiltrated plants, genomic DNA was extracted from leaves, and PCR amplification was performed using pTRV2-specific primers to confirm successful vector introduction. Plants yielding the expected bands size were confirmed to carry the recombinant vectors. Subsequently, qRT-PCR was conducted to assess silencing efficiency by measuring the expression levels of the target genes (*NtLBD4* and *NtLBD31* in tobacco; *SmLBD19* and *SmLBD48* in eggplant) in plants confirmed to carry the viral vector. The corresponding primers are list in **Supplementary Table S2**.

### 2.8 Stress treatment and quantitative real-time PCR analysis

The successfully established plants with uniform growth were divided into three groups for distinct stress treatments: (1) foliar spraying with 100 μmol/L ABA, (2) low-temperature exposure at 5 °C, and (3) root wounding followed by soil drenching with 30 mL of a *Ras* suspension (10^8^ CFU/mL in sterile water). Plants carried the pTRV2 vector were used as the control for each treatment. Plants subjected to ABA or low-temperature stress were sampled at 0, 3, 6, and 12 h post-treatment. For pathogen challenge, plants were maintained in a growth chamber (30 °C, 80% relative humidity, 12 h light/dark cycle), and leaf samples were collected at 0, 1, 3 d post-inoculation. Each sample had three biological replicates, with three individual plants per replicate, totaling 36 plants per treatment. All samples were immediately frozen in liquid nitrogen and stored at -80 °C. Subsequently, qRT-PCR was performed to detect the expression levels of the target genes at the sampling time points.

Total RNA was extracted using the Hipure Plant RNA Mini Kit (AG, Shanghai), and first-strand cDNA was synthesized with the SMART cDNA Synthesis Kit (AG). The expression level of selected *LBD* genes was detected by quantitative real-time PCR (qRT-PCR) using SYBR Green qPCR premix (AG). The actin genes were used as internal references for qRT-PCR normalization in tobacco and eggplant, and all primers were designed using Primer 5 software (**Supplementary Table S3**). The relative expression levels were calculated using the 2^−ΔΔCt^ method (Livak and Schmittgen, 2001). All qRT-PCR reactions were performed in three technical replicates.

### 2.9 Quantification of *Ras* abundance in soil

The population density of *Ras* in rhizosphere soil was determined using a serial-dilution drop-plating method. The experimental procedure and calculation of bacterial abundance were performed with reference to a previously described method (U.S. Food and Drug Administration, 2025; Sofo and Ricciuti, 2019; He et al., 2023). After the appearance of disease symptoms, three symptomatic tobacco plants were randomly selected from each treatment and combined to form one composite rhizosphere-soil sample. Loosely adhering soil was removed by gently shaking the roots, and the soil remaining attached to the roots at a soil depth of approximately 2–5 cm was collected and pooled. Three independent composite samples, comprising a total of nine plants per treatment, were prepared as biological replicates. The collected soil samples were passed through a 2 mm sieve. An aliquot of each sample was oven-dried at 105 °C to a constant weight to determine the dry-to-fresh weight ratio, which was subsequently used to express bacterial abundance on a dry-soil-weight basis. The remaining fresh soil was used to prepare serial dilutions, and 20 μL aliquots of the appropriate dilutions were drop-plated onto triphenyl tetrazolium chloride (TTC) agar plates. Colonies displaying the characteristic pale-pink center on TTC medium were presumptively identified as *Ras* colonies and counted. Because the colony centers gradually turned red with prolonged incubation, colonies were evaluated before excessive color development. *Ras* abundance was expressed as colony-forming units per gram of dry soil (CFU g^-1^ dry soil).

### 2.10 Data analysis

Statistical analysis was performed using SPSS Statistics 27, GraphPad Prism 10, and Microsoft Excel. All quantitative data are presented as mean ± standard deviation (SD). The effects of the two experimental factors and their interaction were evaluated using two-way analysis of variance (ANOVA). When significant effects were detected, multiple comparisons were performed using Duncan’s multiple range test at *p* < 0.05. Figures were prepared and assembled using Adobe Illustrator 2020 and Adobe Photoshop CC 2019.

## 3 Results

### 3.1 Characterization of *LBD* genes in tobacco and eggplant

A total of 106 *NtLBD* genes (*NtLBD1*-*NtLBD106*) and 50 *SmLBD* genes (*SmLBD1*-*SmLBD50*) were identified in tobacco and eggplant, respectively (**Supplementary Tables S4**, **S5**). Protein length varied widely across the tobacco *LBD* family, ranging from 60 aa (*NtLBD7*) to 1518 aa (*NtLBD14*), with most members falling between 100 and 300 aa. Corresponding molecular weights spanned from 6.6 kDa (*NtLBD7*) to 170 kDa (*NtLBD14*). The isoelectric point (pI) of NtLBD proteins also showed considerable diversity, from 4.14 (*NtLBD76*) to 9.88 (*NtLBD60*), suggesting varied charge states under physiological conditions. Hydropathy analysis revealed that only three members (NtLBD11, NtLBD82, NtLBD92) displayed positive GRAVY values, while the rest were negative, indicating that the majority are hydrophilic. In eggplant, SmLBD proteins exhibited a more compact size distribution, primarily between 100 and 300 aa, with the shortest being SmLBD1 (73 aa) and the longest SmLBD10 (367 aa). Molecular weights ranged from 8.5 kDa to 40.8 kDa. The pI values of SmLBDs mirrored the broad distribution observed in tobacco, varying from 4.19 (SmLBD34) to 9.71 (SmLBD36). Similarly, nearly all members (except SmLBD49) showed negative GRAVY values, confirming a predominantly hydrophilic nature for the SmLBD family.

### 3.2 *Cis*-regulatory element analysis of *LBD* genes

The 2 kb upstream sequences from the transcription start site of *NtLBDs* and *SmLBDs* were extracted to analyze their *cis*-acting regulatory elements in the promoter regions. Comparative analysis revealed striking similarities in the *cis*-acting elements between these two *Solanaceae* species (**Supplementary Figures 1**, **2**). A total of 39 cis-acting elements were identified and classified into six categories, including light-responsive elements, hormone-responsive elements, stress response-related elements, developmental-specific expression elements, site-associated *cis*-acting elements, and others. Of these, light-responsive *cis*-elements were highly abundant in both tobacco and eggplant, accounting for 49.65% and 52.96% of all identified cis-elements, respectively. Interestingly, All identified *NtLBD* and *SmLBD* gene promoters contained ABA responsive elements, suggesting a role in ABA mediated regulation. Additionally, a substantial proportion of promoters harbored elements responsive to gibberellin (GA), salicylic acid (SA), and auxin (IAA), implying involvement in hormone signaling and plant developmental processes. Nearly half of the *LBDs* contained MYB transcription factor binding sites, indicating potential direct transcriptional regulation by MYB factors, which may influence ion channel activity and signal transduction pathways.

### 3.3 Phylogenetic analysis of NtLBDs and SmLBDs proteins

To elucidate the evolutionary relationships of the *LBD* gene family, a phylogenetic tree was constructed using *LBD* protein sequences from tobacco (106), eggplant (50), and *Arabidopsis thaliana* (43). Based on previous studies, these LBD proteins were classified into two main classes (Class I and Class II), with Class I further subdivided into subgroups Ia-Id; Class II into subgroups IIa-IIb (**Figure 1**) (Mangeon et al., 2012). In tobacco, Class I accounted for 83.96% (89 members) of all NtLBDs, with subgroup Ia being the largest (25 members). Class II contained only 15 members (14.15%). Similarly, in eggplant, Class I comprised 86.00% (43 members) of SmLBDs, with subgroup Ia being most prominent (14 members), while Class II included 7 members (14.00%). Notably, three LBD proteins (NtLBD60, NtLBD95, and SmLBD46) did not cluster with any subgroup. These results indicate that the *LBD* gene family has remained structurally conserved during evolution, highlighting their fundamental and indispensable roles in plant biology.

**Figure 1:**
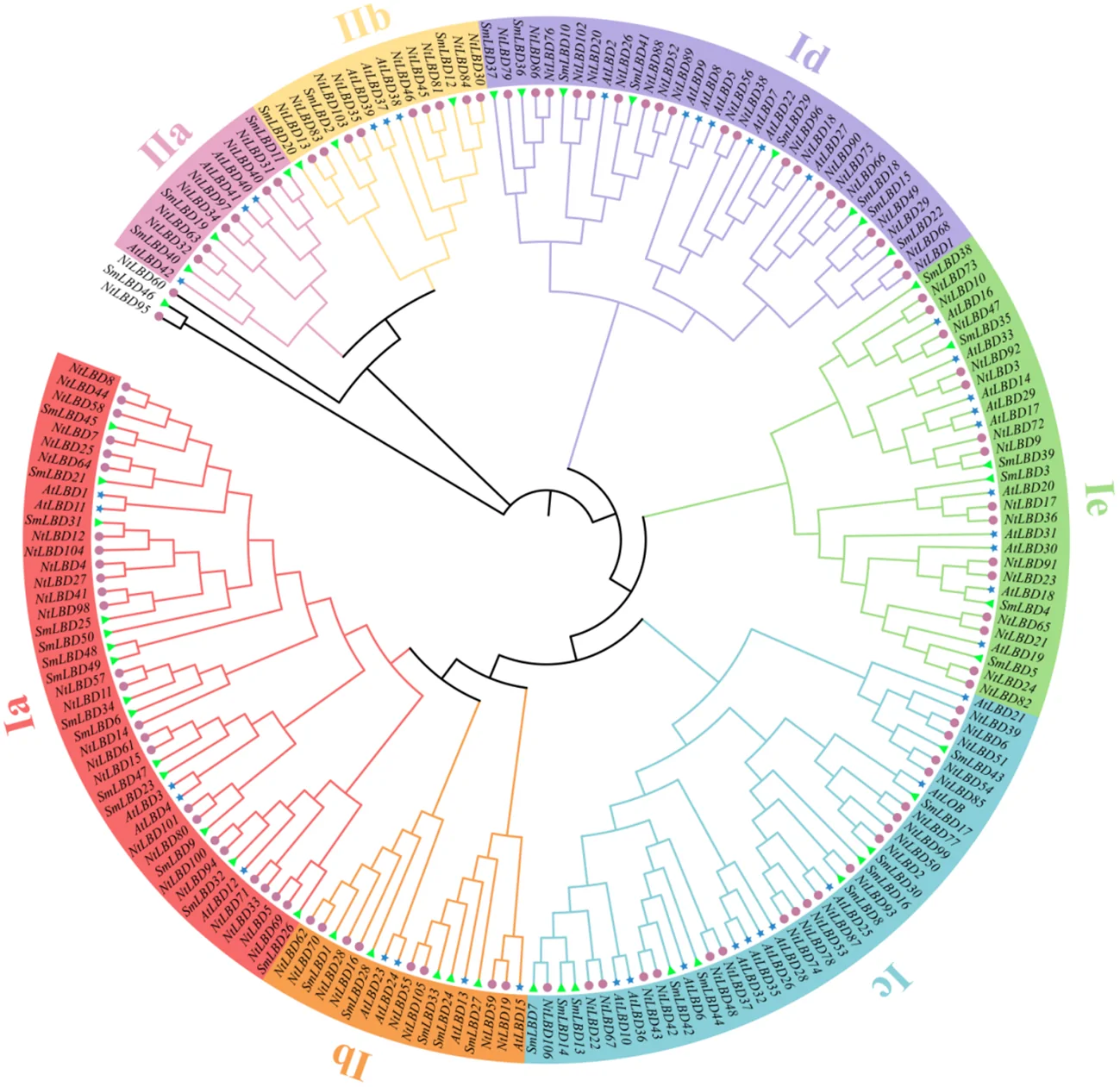
Phylogenetic tree of LBD proteins from tobacco, eggplant, and *Arabidopsis thaliana*. Protein names are abbreviated with species prefixes (At, *Arabidopsis thaliana*; Nt, *Nicotiana tabacum*; Sm, *Solanum melongena*). Branches are color-coded by species: AtLBD (blue star), NtLBD (purple circle), and SmLBD (green triangle).

### 3.4 Chromosome distribution and collinearity analysis of *LBD* genes

Among the 106 *NtLBD* genes, 51 members are unevenly distributed across the 17 tobacco chromosomes, while the remaining 55 members reside on unassigned scaffolds (**Figure 2A**). Notably, chromosome Nt17 harbors the highest number of *NtLBDs* (8), followed by chromosome Nt19 (7). In eggplant, all 50 *SmLBD* members are distributed across 11 chromosomes, with chromosomes E06 (12 genes) and E02 (10 genes) harboring the majority, most of which are located near chromosome termini (**Figure 2B**). Only one *SmLBD* gene is present on chromosomes E04 and E07.

**Figure 2:**
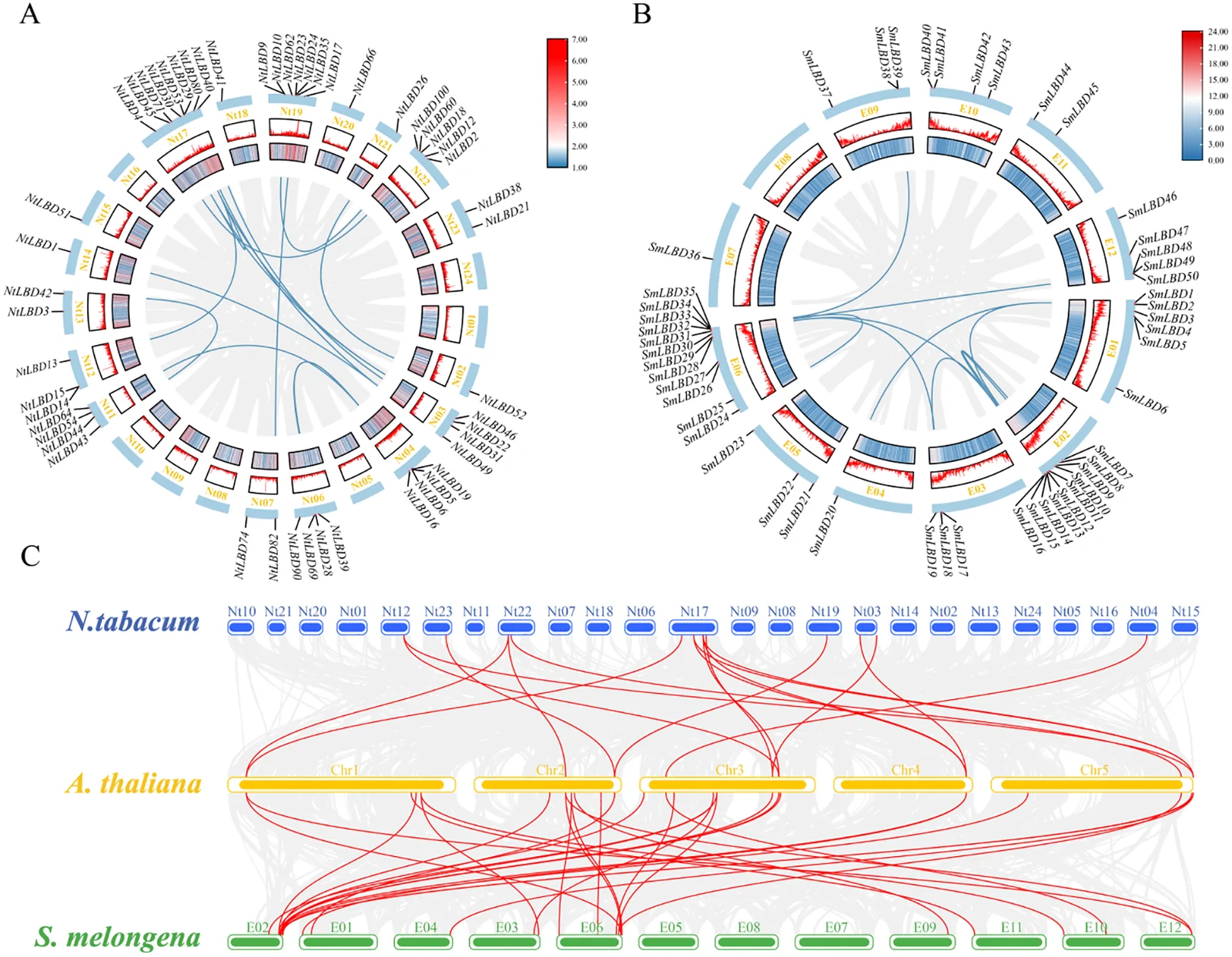
Chromosome distribution and collinearity analysis of *LBD* genes. **(A)** Chromosome locations and inter-chromosomal associations of *NtLBD* genes in tobacco. The blue line indicates the collinearity relationship of *NtLBDs*. **(B)** Chromosome locations and inter-chromosomal associations of *SmLBD* genes in eggplant. The blue line indicates the collinearity relationship of *SmLBDs*. **(C)** Collinearity analysis of *LBD* genes among tobacco, eggplant, and *Arabidopsis thaliana*.

To elucidate the evolutionary mechanisms underlying *LBD* family expansion, gene duplication events were analyzed. *NtLBD* family contains 15 segmental duplication gene pairs and 1 tandem duplication gene pair, while the *SmLBD* family contains 11 segmental duplication gene pairs and 2 tandem duplication gene pairs (**Figures 2A, B**), suggesting that segmental duplication represents a major mechanism underlying *LBD* family expansion in both species (**Supplementary Table S8**). Comparative synteny analysis using MCScanX identified 17 syntenic *LBD* gene pairs between tobacco and *Arabidopsis thaliana* and 31 pairs between eggplant and *Arabidopsis thaliana* (**Figure 2C**; **Supplementary Table S9**). Among these, tobacco chromosome Nt17 and eggplant chromosome E02 harbored the largest number of syntenic *LBD* pairs, suggesting that the corresponding genomic regions and their resident LBD genes have been relatively conserved during evolution.

### 3.5 Expression profiling of *LBDs* in different tissues

To characterize the tissue-specific expression patterns of *LBD* genes, transcriptome data from root, stem, leaf, and flower tissues of tobacco and eggplant were analyzed. Genes with low expression levels (PFKM < 0.5) were excluded to improve analytical reliability. Expression heatmaps were generated for 40 *NtLBDs* and 24 *SmLBDs* (**Supplementary Figure 3**). In tobacco, *NtLBDs* were predominantly expressed in roots and stems, with considerable variation in expression levels among family members. Nine genes, including *NtLBD20*, *NtLBD30*, *NtLBD31*, *NtLBD34*, *NtLBD40*, *NtLBD27*, *NtLBD4*, *NtLBD84*, and *NtLBD97*, exhibited high expression in both root and stem tissues. Notably, *NtLBD31* showed strong expression in roots and stems but low expression in leaves, indicating a potential tissue-specific function in plant development. *SmLBD* genes also displayed diverse spatial expression patterns. For instance, genes such as *SmLBD2*, *SmLBD19*, *SmLBD48*, and *SmLBD12* maintained high expression levels across all four tissues, while others like *SmLBD24*, *SmLBD40*, exhibited high expression in roots, with minimal expression elsewhere. These results highlight both conserved broad-expression profiles and tissue-specific regulatory roles within the *LBD* family, pointing to functional diversification among its members.

### 3.6 Subcellular localization of *NtLBD4*/*31* and *SmLBD19*/*48*

Subcellular localization prediction results suggested that the majority of *NtLBD* and *SmLBD* proteins were localized in the nucleus. To verify this experimentally, we selected *NtLBD4* and *NtLBD31* in tobacco and *SmLBD19* and *SmLBD48* in eggplant for further functional validation based on their tissue-specific expression profiles and phylogenetic classification. Transient expression assays in K326 leaf epidermal cells were performed to examine the subcellular localization of these proteins. Confocal microscopy analysis revealed that all four GFP-fused proteins co-localized completely with a nuclear marker (NLS-mCherry), confirming their nuclear localization (**Figure 3**). Notably, NtLBD31 also displayed clear membrane-associated signals, suggesting potential functional divergence from strictly nuclear proteins.

**Figure 3:**
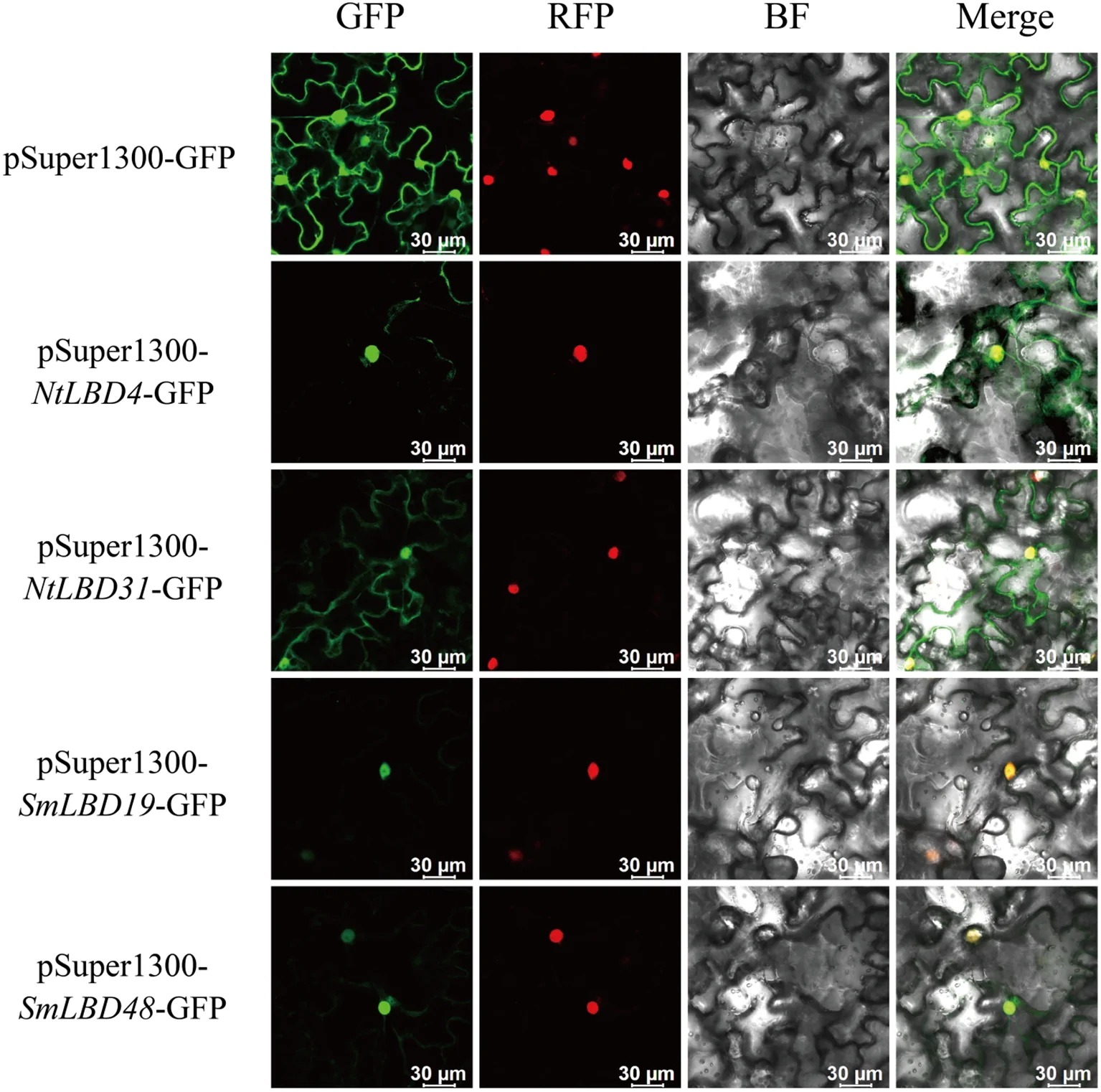
Subcellular localization of *NtLBD4*, *NtLBD31*, *SmLBD19*, and *SmLBD48*. The constructs pSuper1300-*NtLBD4*-GFP, pSuper1300-*NtLBD31*-GFP, pSuper1300-*SmLBD19*-GFP, pSuper1300-*SmLBD48*-GFP, and pSuper1300-GFP (control) were transiently expressed in epidermal cells of tobacco cultivar K326 via *Agrobacterium*-mediated transformation. Fluorescence signals were observed by confocal microscopy 48 h after transformation. Scale bar = 30 µm.

### 3.7 Validation of the VIGS system

To verify the successful establishment of the VIGS system, GFP fluorescence was monitored in infiltrated tobacco and eggplant plants. Clear green fluorescence was observed in the infiltrated leaves under UV illumination at 10–15 days post-infiltration, indicating successful delivery and expression of the recombinant TRV constructs (**Supplementary Figure 4A**). The transcript abundance of the intended target genes was subsequently assessed using gene-specific qRT-PCR primers. Compared with the corresponding empty-vector controls, the transcript levels of *NtLBD4* and *NtLBD31* in tobacco and *SmLBD19* and *SmLBD48* in eggplant were significantly reduced in plants infiltrated with the respective pTRV-*LBD* constructs (**Supplementary Figure 4B**). These results confirmed reduced accumulation of the intended target-gene transcripts. However, because the expression of closely related homologous genes was not examined, possible co-silencing of such genes cannot be excluded.

### 3.8 *LBD* gene silencing rewires the ABA response network and triggers synergistic and compensatory effect

Previous studies have shown that *LBD* family members play pivotal roles in plant responses to biotic and abiotic stresses. For example, overexpression of *MdLBD37* (Class IIa) alleviates ABA-mediated growth inhibition in apple (Li et al., 2025), while cold stress induces *MaLBD5* (Class Ia) expression in banana, which modulates jasmonic acid biosynthesis to enhance cold tolerance (Ba et al., 2016). Conversely, silencing *GhLBD41* compromises cotton resistance to *Verticillium dahliae* (Hu et al., 2024). In this study, *NtLBD4, NtLBD31*, *SmLBD48, and SmLBD19*, which phylogenetically cluster with the above genes in Class Ia/IIa (**Figure 1**), displayed high expression levels and broad tissue distribution (**Supplementary Figure 3**). This suggests that these four genes may likewise perform prominent, and potentially conserved, functions in stress-related physiological processes.

To elucidate the regulatory roles of *LBD* members under ABA stress, we employed pTRV2-mediated VIGS to generate gene-silenced plants in tobacco and eggplant, and then examined expression changes in the target genes, ABA pathway-related genes, and clustered members after treatment with ABA. qRT-PCR analysis showed that transcript levels of *NtLBD4* and *NtLBD31* were significantly reduced in pTRV2-*NtLBD4* and pTRV2-*NtLBD31* plants, respectively, confirming effective silencing (**Figure 4**). Compared with the control pTRV2, the expression levels of the ABA pathway-related genes *NtAREB*, *NtNCED3*, *NtPYL8*, and *NtSnRK2.6* in pTRV2-*NtLBD4* plants were generally decreased, indicating that loss of *NtLBD4* attenuates the molecular responses of modules associated with ABA signal transduction and biosynthesis. Concurrently, the ABA-induced temporal expression profile of *NtLBD4* was altered, showing delayed induction and a more pronounced expression peak at 12 h. Although five genes (*NtLBD12, NtLBD27, NtLBD41, NtLBD98, and NtLBD104*) clustered with *NtLBD4* typically exhibited an “upregulation followed by downregulation” pattern, their overall expression levels remained lower than those in the control plants (**Figure 4A**), suggesting that they may act coordinately with *NtLBD4* during ABA responses. Meanwhile, in *NtLBD31*-silenced (pTRV2-*NtLBD31*) plants, the response to ABA stress was attenuated in magnitude but accelerated in timing: the expression peak shifted from 12 h in the control to 3 h. These observations indicate that *NtLBD31* modulates both the intensity and dynamics of ABA-induced transcriptional responses. In addition, expression levels of its clade members *NtLBD32* and *NtLBD34* were significantly elevated (**Figure 4B**), suggesting partial functional compensation in response to *NtLBD31* suppression.

**Figure 4:**
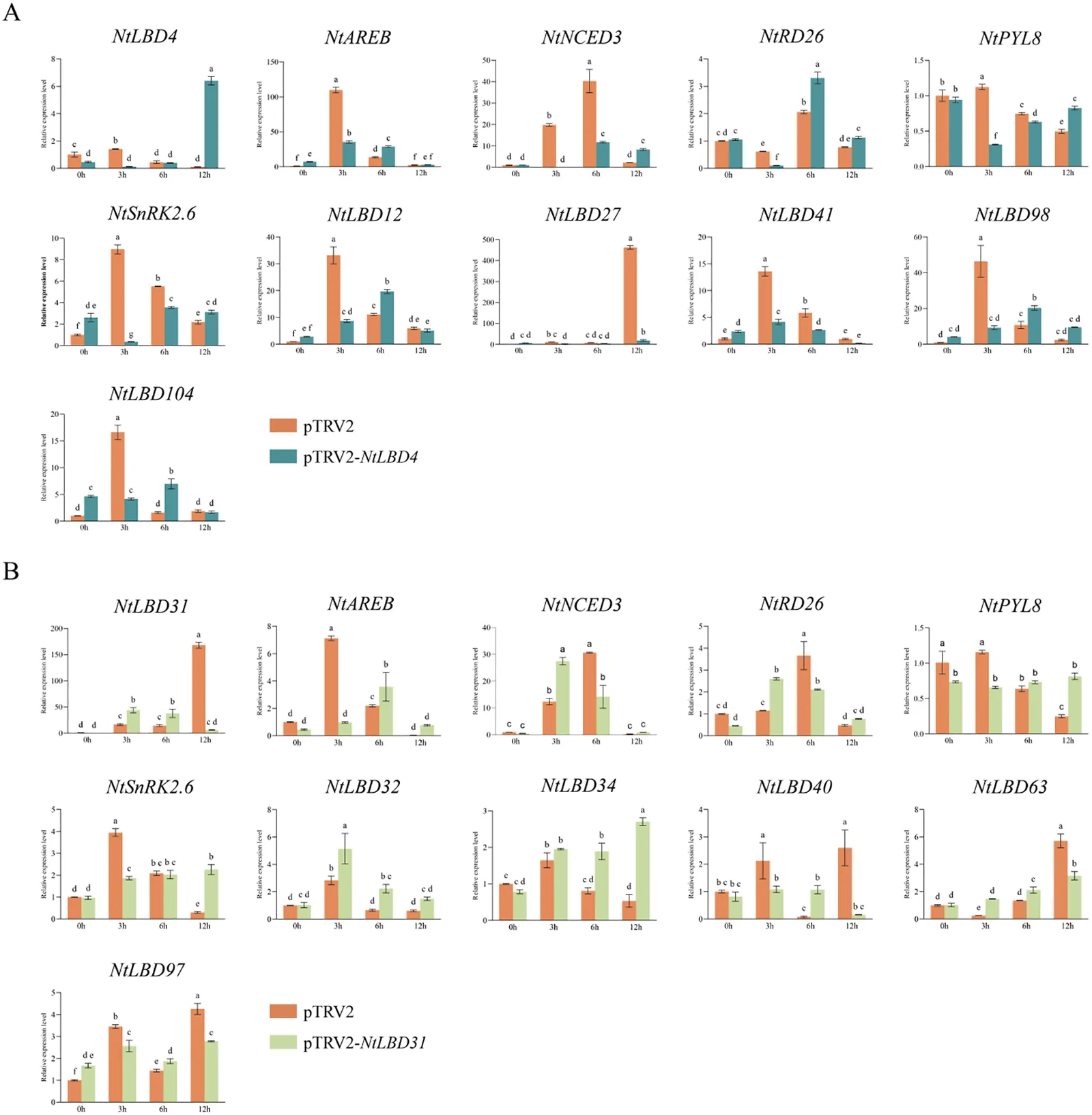
Expression profiles of *NtLBD* genes and ABA pathway-related genes under ABA stress. **(A)** Expression of *NtLBD4* and its clade members, along with key ABA-related genes in control (pTRV2) and *NtLBD4-*silenced (pTRV2-*NtLBD4)* tobacco plants. **(B)** Expression of *NtLBD31* and its clade members, along with key ABA-related genes, in control (pTRV2) and *NtLBD31*-silenced (pTRV2-*NtLBD31*) tobacco plants. Data are presented as mean ± SD (n = 3). Statistical differences were analyzed using two-way ANOVA followed by Duncan’s multiple range test. Different lowercase letters above the bars indicate significant differences among treatment combinations at *p* < 0.05. Bars sharing at least one common lowercase letter are not significantly different, whereas bars with no letters in common are significantly different. This notation reflects the results of *post hoc* multiple comparisons across all treatment groups within each image.

Effective silencing of *SmLBD19* and *SmLBD48* by VIGS was confirmed by their significantly reduced expression levels (**Figure 5**). Compared with the control pTRV2, silencing of *SmLBD19* led to a general up-regulation of ABA biosynthesis-related genes (*SmNCED1*, *SmGSTU10*, *SmDHNX1*, and *SmTAS14*) and its clade-related members *SmLBD11* and *SmLBD40* under ABA treatment (**Figure 5A**). Similarly, in *SmLBD48*-silenced plants, *SmLBD48* itself remained strongly ABA-inducible (**Figure 5B**). Furthermore, most ABA biosynthesis-related genes and clade-related members (except for *SmLBD13*) were upregulated at different post-treatment stages, highlighting functional divergence among Class I *SmLBDs* in ABA pathways.

**Figure 5:**
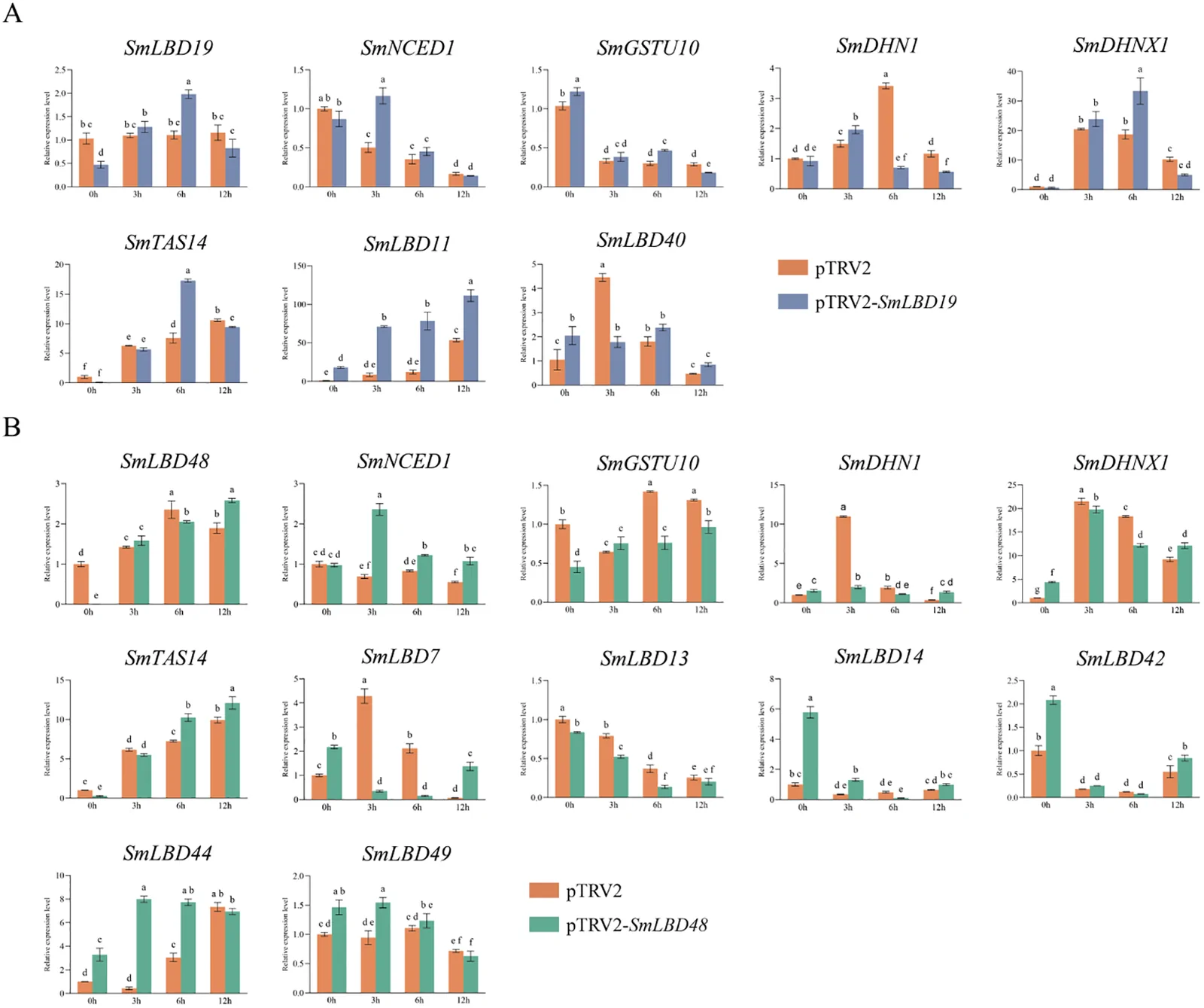
Expression profiles of *SmLBD* genes and ABA pathway-related genes under ABA stress. **(A)** Expression of *SmLBD19* and its clade members, along with key ABA-related genes in control (pTRV2) and *SmLBD19*-silenced (pTRV2-*SmLBD19*) eggplant plants. **(B)** Expression of *SmLBD48* and its clade members, along with key ABA-related genes, in control (pTRV2) and *SmLBD48*-silenced (pTRV2-*SmLBD48*) eggplant plants. Data are presented as mean ± SD (n = 3). Statistical differences were analyzed using two-way ANOVA followed by Duncan’s multiple range test. Different lowercase letters above the bars indicate significant differences among treatment combinations at *p* < 0.05. Bars sharing at least one common lowercase letter are not significantly different, whereas bars with no letters in common are significantly different. This notation reflects the results of *post hoc* multiple comparisons across all treatment groups within each image.

### 3.9 Silencing *LBD* genes enhanced cold tolerance in tobacco and eggplant

To investigate the potential roles of tobacco *NtLBD4*/*31* and eggplant *SmLBD19*/*48* in cold-stress responses, confirmed gene-silenced tobacco and eggplant plants were transferred to a growth chamber maintained at 5 °C, and phenotypic changes were monitored in parallel with transcriptional responses of the relevant genes. At the early stage of cold treatment (3 h), no obvious phenotypic differences were observed among the tobacco seedlings. However, after 6 h of exposure, the pTRV2 control plants exhibited leaf wrinkling and pronounced wilting, whereas the pTRV2-*NtLBD4/31*-silenced plants showed only slight drooping. By 12 h, the pTRV2-*NtLBD4/31* plants had largely returned to a normal phenotype, while the control plants still displayed mild wilting (**Figure 6A**). These observations indicate that silencing *NtLBD4* and *NtLBD31* enhances cold tolerance in tobacco.

**Figure 6:**
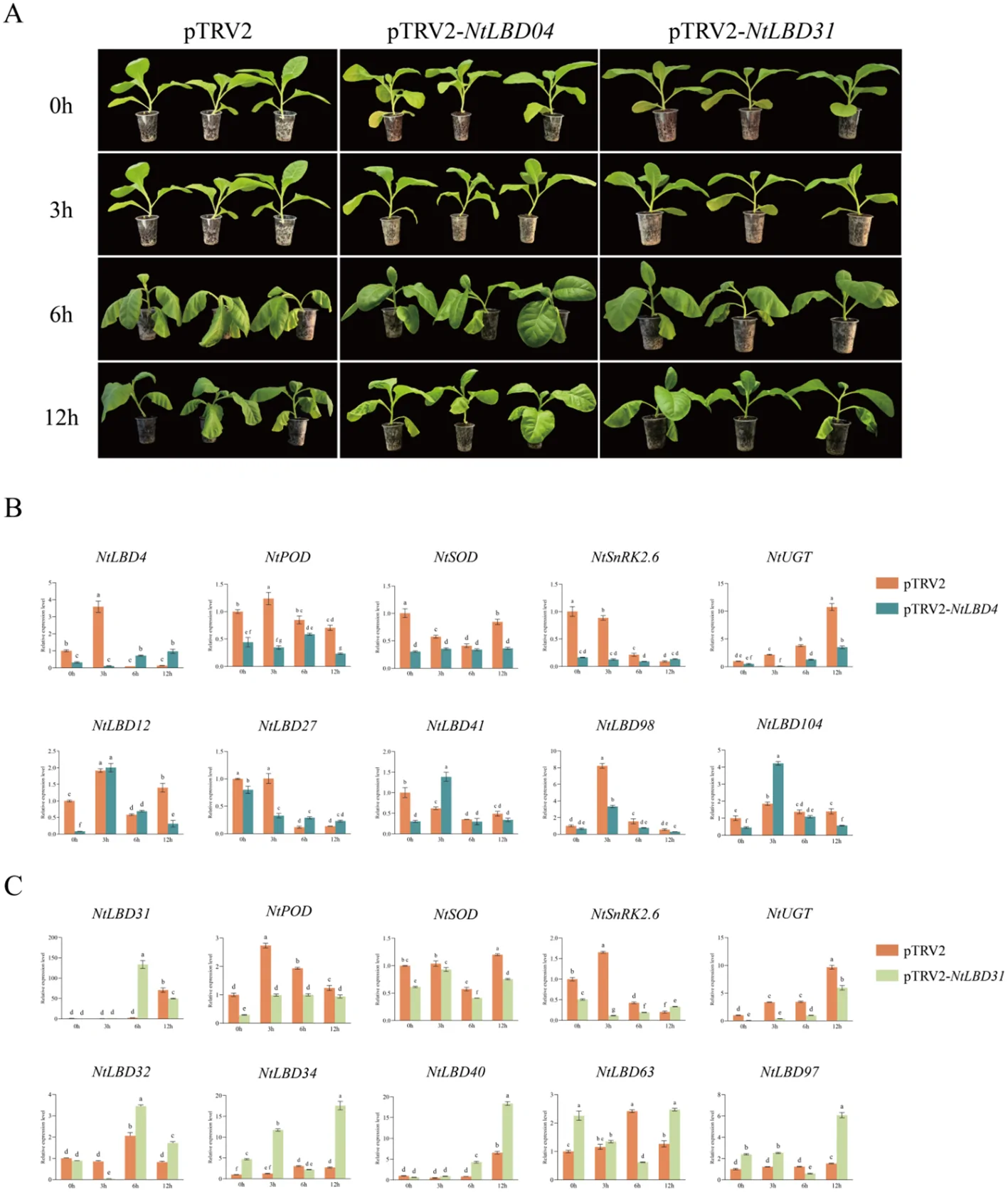
Phenotypic changes and molecular responses of *NtLBD*-silenced tobacco plants to cold stress. **(A)** Phenotype of control (pTRV2) and VIGS*-*silenced (pTRV2-*NtLBD4/31)* plants under cold treatment; **(B)** Expression profiles of key cold-responsive genes and target *NtLBD* clade members in control vs. *NtLBD4*-silenced (pTRV2-*NtLBD4*) plants; **(C)** Expression profiles of key cold-responsive genes and target *NtLBD* clade members in control vs. *NtLBD31*-silenced (pTRV2-*NtLBD31*) plants. Data are presented as mean ± SD (n = 3). Statistical differences were analyzed using two-way ANOVA followed by Duncan’s multiple range test. Different lowercase letters above the bars indicate significant differences among treatment combinations at *p* < 0.05. Bars sharing at least one common lowercase letter are not significantly different, whereas bars with no letters in common are significantly different. This notation reflects the results of *post hoc* multiple comparisons across all treatment groups within each image.

Compared with the pTRV2 control, the expression levels of stress-responsive genes (*NtPOD*, *NtSOD*, *NtSnRK2.*6, and *NtUGT*) were consistently lower in pTRV2-*NtLBD4/31*-silenced plants under low-treatment (**Figures 6B, C**). This observation suggests that silencing *NtLBD4/31* likely attenuates the cold-induced activation of the associated pathways, thereby leading to reduced expression of these genes. Furthermore, while the cold response in pTRV2-*NtLBD4* plants appeared delayed, expression of clade members *NtLBD27*, *NtLBD41*, and *NtLBD104* eventually exceeded control levels during the later stages of cold treatment (**Figure 6B**), suggesting a temporally shifted and possibly intensified compensatory response. In contrast, pTRV2-*NtLBD31* plants exhibited an earlier (6 h) and substantially stronger cold-induced response compared to the pTRV2 control. Their clade members (*NtLBD32/34/40/63/97*) displayed distinct temporal expression profiles with an overall upward trend under cold stress (**Figure 6C**), suggesting compensatory regulation or transcriptional rewiring following *NtLBD31* silencing.

In eggplant, seedlings of both the pTRV2 control and the pTRV2-*SmLBD19* group showed mild wilting after 3 h of low-temperature treatment, with wilting severity increasing over time. By contrast, seedlings of the pTRV2-*SmLBD48* group maintained good physiological performance under cold conditions and displayed no obvious wilting until 12 h, when only slight symptoms appeared. This phenotype indicates that silencing *SmLBD48* enhances cold tolerance in eggplant (**Figure 7A**).

**Figure 7:**
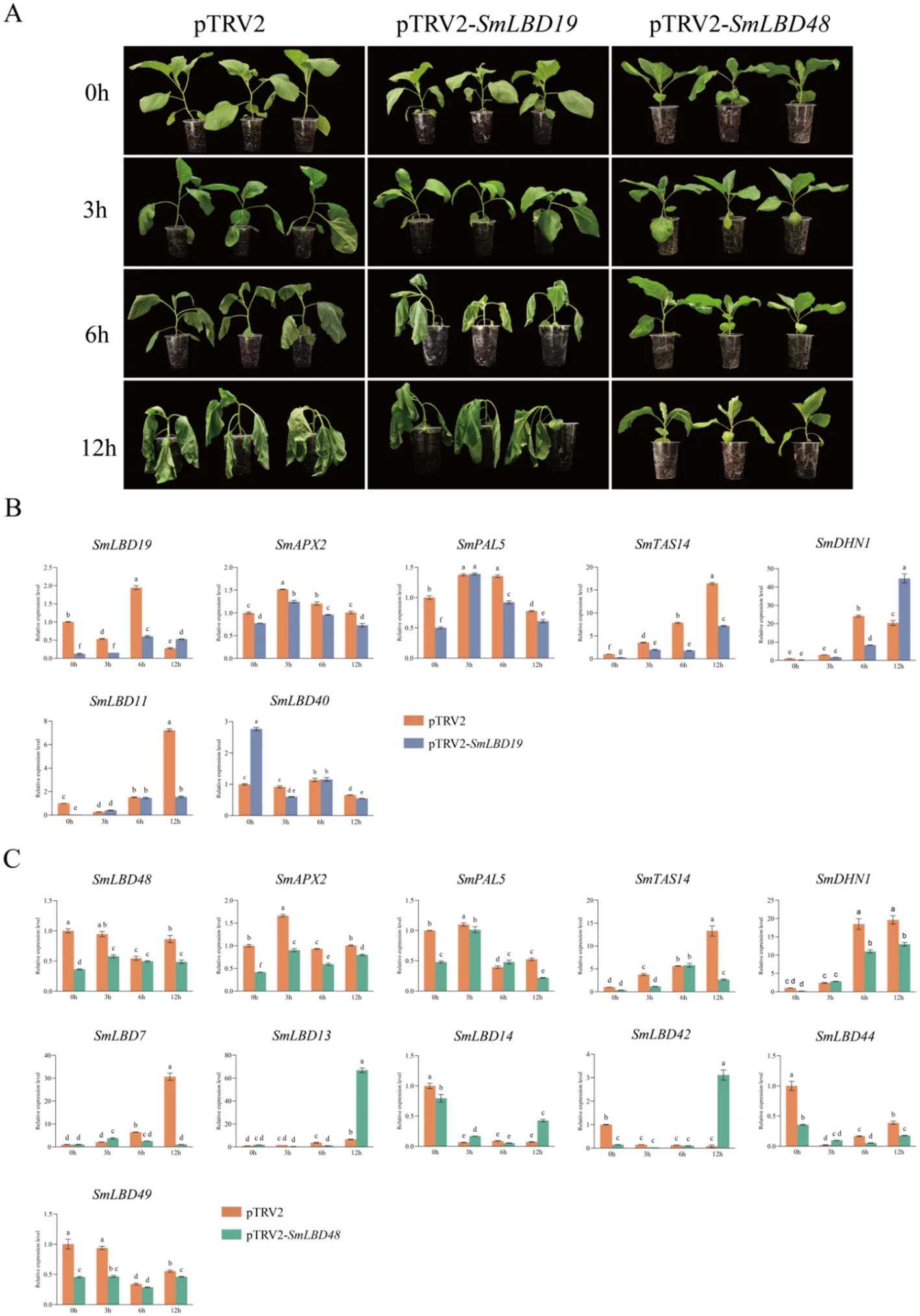
Phenotypic changes and molecular responses of *SmLBD*-silenced eggplant plants to cold stress. **(A)** Phenotype of control (pTRV2) and VIGS*-*silenced (pTRV2-*SmLBD19/48)* plants under cold treatment; **(B)** Expression profiles of key cold-responsive genes and target *SmLBD* clade members in control vs. *SmLBD19*-silenced (pTRV2-*SmLBD19*) plants; **(C)** Expression profiles of key cold-responsive genes and target *SmLBD* clade members in control vs. *SmLBD48*-silenced (pTRV2-*SmLBD48*) plants. Data are presented as mean ± SD (n = 3). Statistical differences were analyzed using two-way ANOVA followed by Duncan’s multiple range test. Different lowercase letters above the bars indicate significant differences among treatment combinations at *p* < 0.05. Bars sharing at least one common lowercase letter are not significantly different, whereas bars with no letters in common are significantly different. This notation reflects the results of *post hoc* multiple comparisons across all treatment groups within each image.

To further characterize the transcriptional response under cold treatment, qRT-PCR was performed on cold-responsive genes and *LBD* genes that cluster phylogenetically with *SmLBD19* and *SmLBD48* in the same subgroups (**Figures 7B, C**). Compared to pTRV2 control, the cold-responsive genes *SmAPX2*, *SmPAL5*, and *SmTAS14* were down-regulated to varying degrees in pTRV2-*SmLBD19* plants during cold exposure, whereas *SmDHN1* transcript levels increased markedly at 12 h. Concurrently, the clade-related gene *SmLBD11* showed a weaker cold-induced response in pTRV2-*SmLBD19* plants than in the control, while *SmLBD40* exhibited a stronger response (**Figure 7B**). In the pTRV2-*SmLBD48* group (**Figure 7C**), the overall expression levels of cold-responsive genes (*SmAPX2*, *SmPAL5*, *SmTAS14*, and *SmDHN1*) were markedly lower than those in the control, yet their temporal patterns differed during cold treatment. *SmAPX2* displayed a control-like profile, being rapidly induced at 3 h to a peak and then declining. *SmDHN1* and *SmTAS14* were induced during the mid-to-late stages, whereas *SmPAL5* showed a more pronounced early transient upregulation in the silenced plants followed by a reduction at later time points. Concurrently, the clade-related genes *SmLBD7*, *SmLBD44*, and *SmLBD49* showed a weaker cold-induced response in pTRV2-*SmLBD48* plants than in the control, while *SmLBD13* and *SmLBD42* exhibited a stronger response. This pattern suggests functional divergence and a potential compensatory role between the LBD genes.

### 3.10 *LBD* genes modulate the responses of tobacco and eggplant to *Ras* infection

To investigate the roles of tobacco *NtLBD4* and *NtLBD31* and eggplant *SmLBD19* and *SmLBD48* in defense against bacterial wilt, *Ras* inoculation assays were performed, and disease progression was monitored daily for three consecutive days. Following inoculation, *Ras* population densities in the rhizosphere soil were quantified for all tobacco and eggplant treatment groups using a serial-dilution plating method. *Ras* populations across the treatment groups ranged from 5.6 × 10^8^ to 9.1 × 10^8^ CFU g^-1^ dry soil, indicating successful establishment of the pathogen in the rhizosphere (**Supplementary Figure 5**).

In tobacco (**Figure 8A**), both the empty-vector control plants (pTRV2) and the *NtLBD4* or *NtLBD31* silenced plants developed wilting symptoms following *Ras* inoculation, with symptom severity increasing over time. At 1 dpi, the control plants exhibited slight leaf drooping, whereas more pronounced wilting was observed in the pTRV2-*NtLBD4* and pTRV2-*NtLBD31* groups. By 3 dpi, symptoms had intensified in all groups; however, wilting remained more severe in both gene-silenced groups than in the control group. The high *Ras* population densities detected in the rhizosphere, together with the development of characteristic wilting symptoms, supported the successful establishment of *Ras* infection. Collectively, silencing *NtLBD4* or *NtLBD31* resulted in an earlier onset and greater severity of wilting following *Ras* inoculation, suggesting that both genes positively contribute to tobacco resistance against bacterial wilt.

**Figure 8:**
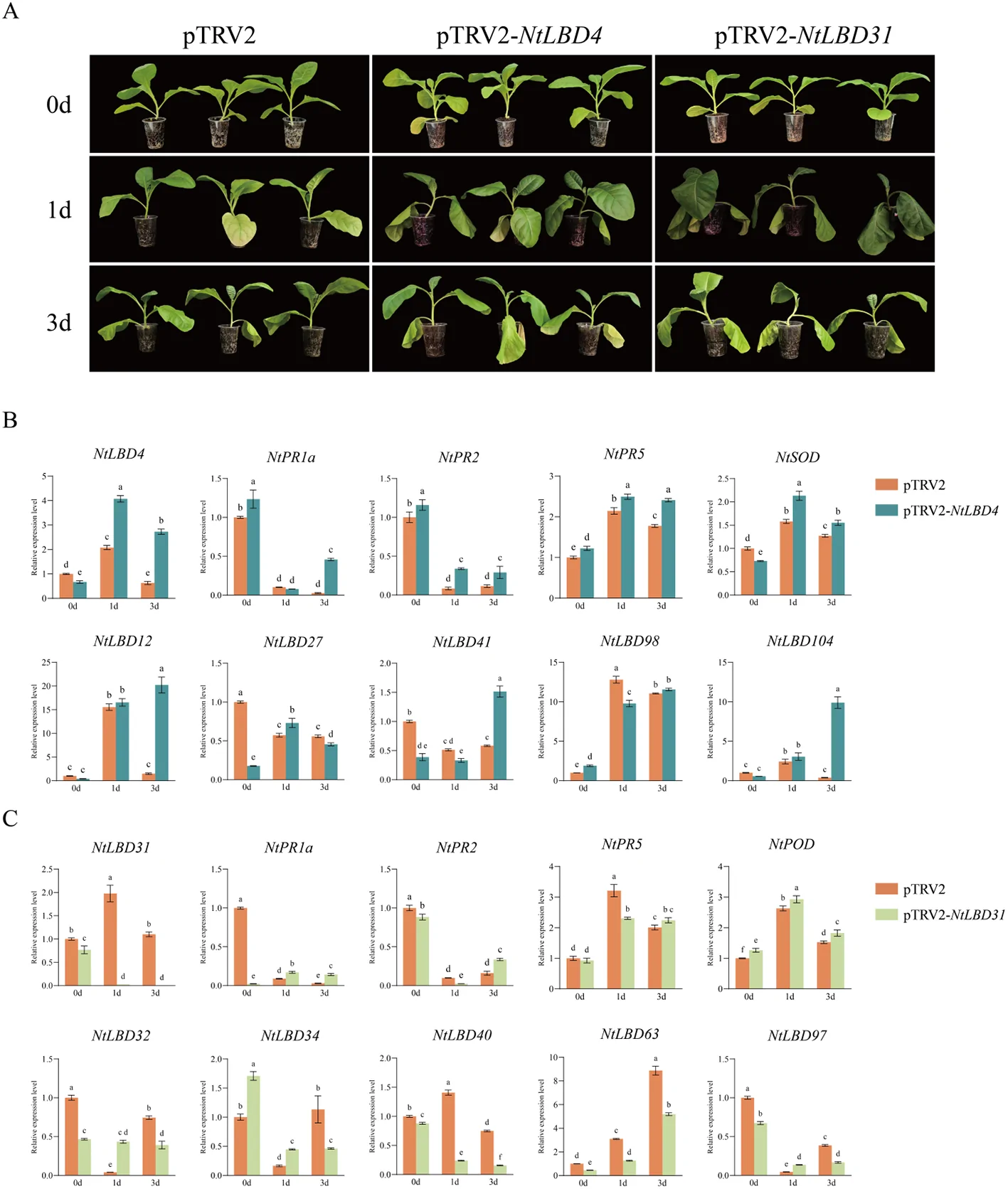
Phenotypic changes and molecular responses of *NtLBD*-silenced tobacco plants to *Ras* infection. **(A)** Phenotype of control (pTRV2) and VIGS*-*silenced (pTRV2-*NtLBD4/31*) plants under *Ras* infection; **(B)** Expression profiles of key stress-responsive genes and target *NtLBD* clade members in control vs. *NtLBD4*-silenced (pTRV2-*NtLBD4*) plants under *Ras* infection; **(C)** Expression profiles of key stress-responsive genes and target *NtLBD* clade members in control vs. *NtLBD31*-silenced (pTRV2-*NtLBD31*) plants under *Ras* infection. Data are presented as mean ± SD (n = 3). Data are presented as mean ± SD (n = 3). Statistical differences were analyzed using two-way ANOVA followed by Duncan’s multiple range test. Different lowercase letters above the bars indicate significant differences among treatment combinations at *p* < 0.05. Bars sharing at least one common lowercase letter are not significantly different, whereas bars with no letters in common are significantly different. This notation reflects the results of *post hoc* multiple comparisons across all treatment groups within each image.

To investigate the roles of tobacco *NtLBD4/31* and eggplant *SmLBD19/48* in defense against bacterial wilt, pathogen *Ras* inoculation assays were conducted, and disease progression was monitored over three consecutive days. In tobacco (**Figure 8A**), both control (pTRV2) and the VIGS-silenced pTRV2-*NtLBD4/31* plants developed wilting symptoms after inoculation, with severity increasing over time. At 1 dpi, control plants showed slight leaf drooping, while wilting was more pronounced in the pTRV2-*NtLBD4* and pTRV2-*NtLBD31* groups. By 3 dpi, symptoms had intensified in all groups; however, overall wilting remained more severe in the pTRV2-*NtLBD4* and pTRV2-*NtLBD31* plants compared to controls. Collectively, *NtLBD4*/*31*-silenced plants displayed an earlier onset and greater severity of wilting under *Ras* stress, implicating both genes in tobacco’s defense response against *Ras* infection.

qRT-PCR analysis revealed distinct expression differences between the control (pTRV2) and the VIGS-silenced pTRV2-*NtLBD4/31* groups. Under pathogen *Ras* infection (**Figure 8B**), expression of *NtLBD4* and dissease-related genes (*NtPR1a*, *NtPR2*, *NtPR5*, and *NtSOD)* were generally upregulated in pTRV2-*NtLBD4* plants compared to the control. Meanwhile, clade-related genes of *NtLBD4* exhibited divergent expression patterns in the *NtLBD4*-silenced background: *NtLBD12/41/104* were significantly induced at 3 dpi, whereas *NtLBD27* and *NtLBD98* showed comparatively modest changes. In contrast, *NtLBD31* silencing resulted in reduced expression of *NtPR1a*, *NtPR2*, and *NtPR5* under pathogen *Ras* infection, while *NtSOD* was slightly upregulated. These findings suggest that *NtLBD31* silencing disrupts the regulation of stress-responsive genes, though the link between these transcriptional changes and disease phenotypes requires further validation. Consistent with this disruption (**Figure 8C**), the induction dynamics of *NtLBD31* clade members were also altered in the *NtLBD31*-silenced background. At 1 dpi, expression of *NtLBD32, NtLBD34*, and *NtLBD97* was significantly higher than in the control, whereas *NtLBD40* and *NtLBD63* were lower. By 3 dpi, all examined clade members were expressed at levels below the control. This pattern may indicate a stage-dependent adjustment of clade-member regulation in response to *NtLBD31* deficiency.

In eggplant infected with *Ras*, seedlings exhibited mild wilting at 1 dpi, which progressed to more severe symptoms by 3 dpi. Compared with the control, both silenced lines displayed overall milder wilting and better plant vigor. The pTRV2-*SmLBD48* treatment showed the most pronounced alleviation, with the lowest overall wilting severity (**Figure 9A**). These results imply that *SmLBD19* and *SmLBD48* likely function as negative regulators in response to *Ras* infection.

**Figure 9:**
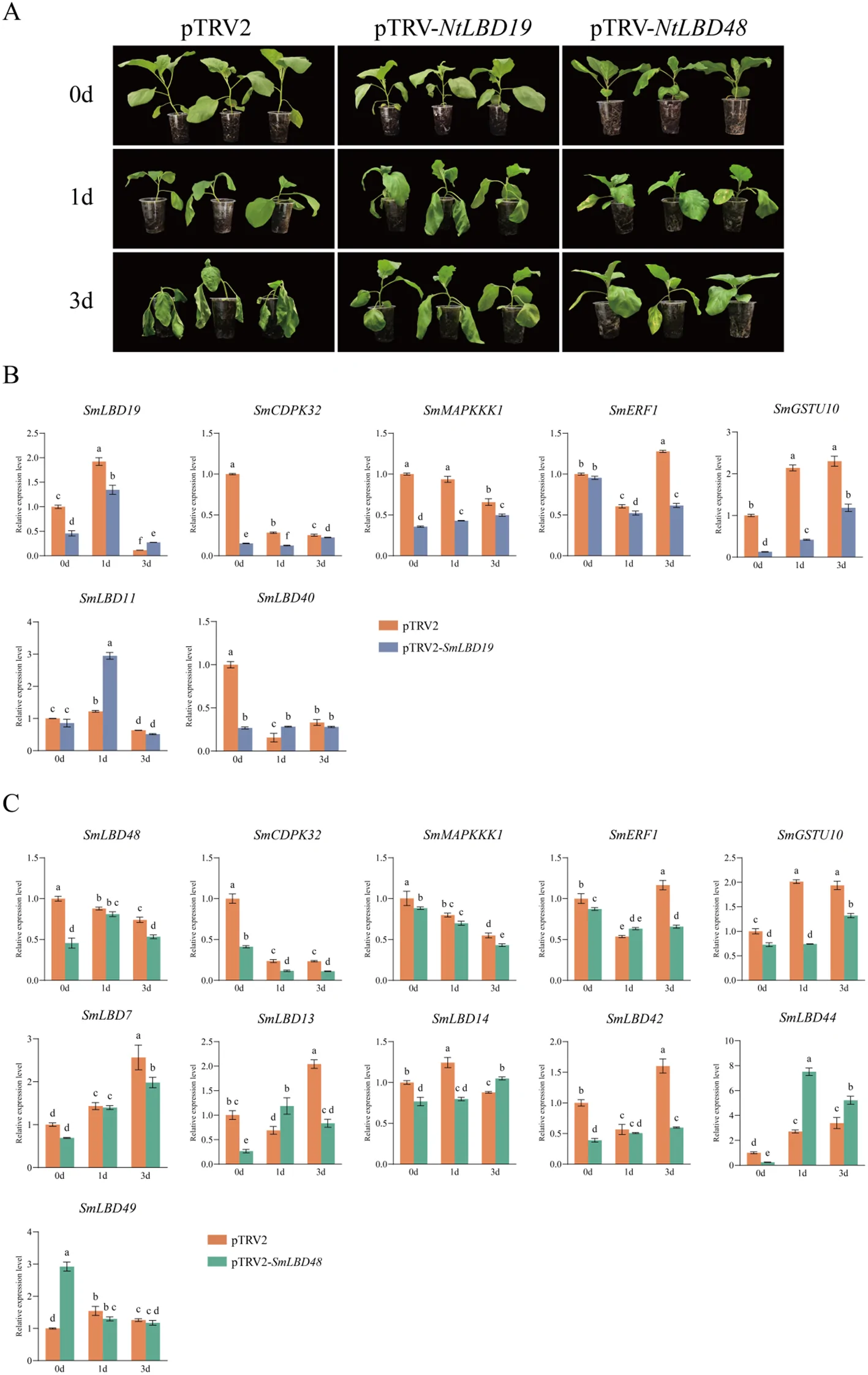
Phenotypic changes and molecular responses of *SmLBD*-silenced eggplant plants to *Ras* infection. **(A)** Phenotypic of control (pTRV2) and VIGS*-*silenced (pTRV2-*SmLBD19/48)* plants under *Ras* infection; **(B)** Expression profiles of key disease-responsive genes and target *SmLBD* clade members in control vs. *SmLBD19*-silenced (pTRV2-*SmLBD19*) plants under *Ras* infection; **(C)** Expression profiles of key disease-responsive genes and target *SmLBD* clade members in control vs. *SmLBD48*-silenced (pTRV2-*SmLBD48*) plants. Data are presented as mean ± SD (n = 3). Data are presented as mean ± SD (n = 3). Statistical differences were analyzed using two-way ANOVA followed by Duncan’s multiple range test. Different lowercase letters above the bars indicate significant differences among treatment combinations at *p* < 0.05. Bars sharing at least one common lowercase letter are not significantly different, whereas bars with no letters in common are significantly different. This notation reflects the results of *post hoc* multiple comparisons across all treatment groups within each image.

In pTRV2-*SmLBD19* plants (**Figure 9B**), the stress-responsive genes *SmCDPK32*, *SmMAPKKK1*, *SmERF1*, and *SmGSTU10* were downregulated to varying degrees after pathogen inoculation compared to the control, indicating that *SmLBD19* silencing suppresses the transcription of genes in stress-response pathways. Meanwhile, the clade-related gene *SmLBD11* exhibited a markedly stronger induction at 1 dpi in the silenced plants than in the control, followed by a decline by 3 dpi, whereas *SmLBD40* maintained an overall lower expression level with attenuated temporal variation in the silenced background. In the pTRV2-*SmLBD48* plants (**Figure 9C**), *SmCDPK32*, *SmMAPKKK1*, *SmERF1*, and *SmGSTU10* were also down-regulated to varying degrees after pathogen inoculation compared to the control. In addition, silencing *SmLBD48* induced differential expression reconfiguration of its clade members. For instance, *SmLBD7* and *SmLBD42* showed weaker responses in pTRV2-*SmLBD48* plants relative to the control, whereas *SmLBD44* and *SmLBD49* exhibited stronger responses. Together, these changes indicate that *SmLBD48* silencing reshapes the temporal expression dynamics of clade-related *LBD* genes.

## 4 Discussion

Members of the *LBD* gene family are widely distributed across diverse plant species. Advances in genome sequencing technologies have greatly facilitated the identification of *LBD* genes in multiple plant genomes, with representative species including *Arabidopsis thaliana* (Shuai et al., 2002), *Populus trichocarpa* (Dang et al., 2024), *Solanum lycopersicum* L (Dong and Manghwar, 2023), and *Physcomitrella patens* (Huang et al., 2020). In this study, a total of 106 *NtLBD* genes in tobacco and 50 *SmLBD* genes in eggplant were identified. These *NtLBD* and *SmLBD* genes could be divided into 2 Classes, of which the Class I contained the most members in tobacco and eggplant. This distribution suggests a significant evolutionary selection favoring the expansion of Class I, similar to patterns observed in model organisms such as *Arabidopsis thaliana* (Shuai et al., 2002) and *Cucumis melo* L (Derelli Tufekci, 2023). Polyploidization and region-specific gene duplication events—including tandem and segmental duplications—are considered major mechanisms driving the expansion of plant gene families (Cannon et al., 2004; Moore and Purugganan, 2003). A total of 15 duplicated gene pairs were identified in the *NtLBDs*, and 11 pairs in the *SmLBDs*. In the comparative collinearity analysis with *Arabidopsis thaliana*, 17 and 31 pairs of syntenic relationships were found for *NtLBD* and *SmLBD*, respectively. The number of members within the same gene family often varies across species, largely driven by gene duplication events that occur at unequal frequencies among different lineages, thereby promoting family expansion as well as functional innovation and environmental adaptation (Domazet-Lošo et al., 2024). Notably, the tobacco genome assembly remains incomplete, and nearly half of the *NtLBD* genes are located on scaffold sequences; this feature may lead to an apparently lower frequency of gene duplication events than that observed in eggplant. In addition, the number of *NtLBD* genes in tobacco is more than twice that in *Arabidopsis thaliana*, a pattern that may be associated with the allotetraploid nature of tobacco (Li et al., 2024b).

Plants are continually exposed to diverse stresses during growth and development, ranging from hormone-signaling fluctuations and abiotic challenges such as low temperature and drought to biotic threats like pathogen infection (Zhu, 2016; Jones and Dangl, 2006; Guan et al., 2023). Such adverse conditions disrupt physiological metabolism and defense regulation, severely hampering plant growth and reducing yield (Savary et al., 2019). Given the persistent and severe losses in crop quality and productivity caused by diseases, mining and exploiting elite stress-resistance genes in plants have become an important strategy for improving crop stress tolerance and ensuring stable yields (Zhao et al., 2022). Accumulating evidence indicates that members of the *LBD* gene family play pivotal roles in plant defense responses in diverse stress-response processes (Guo et al., 2020; Feng et al., 2022; Sun et al., 2025). ABA is a central hormonal signal mediating responses to drought, salinity, low temperature, and other environmental stresses, and it can activate the expression of downstream stress-responsive genes to enhance plant stress tolerance (Cutler et al., 2010). Previous studies have shown that *AtLBD15* enhances drought resistance by positively regulating the ABA signaling pathway (Guo et al., 2020), whereas *ZmLBD35* negatively regulates ABA sensitivity within the same pathway and influences ABA biosynthesis (Feng et al., 2022). In the present study, VIGS targeting *NtLBD4* or *NtLBD31* was associated with significant down-regulation of ABA-responsive genes and altered response dynamics under exogenous ABA treatment, suggesting that these tobacco *LBD* genes may participate in the positive regulation of ABA biosynthesis or signaling. Conversely, VIGS targeting *SmLBD19* or *SmLBD48* in eggplant was associated with a general up-regulation of genes involved in ABA biosynthesis and signaling, suggesting that these genes may participate in the negative regulation of the ABA pathway in this species. The contrasting expression patterns observed in the two species imply that members of the *LBD* gene family may have undergone substantial functional divergence during evolution. Such divergence may be attributable to the subfunctionalization or neofunctionalization of homologous genes, as well as differences in the transcriptional regulatory networks in which LBD proteins operate across species (Conant and Wolfe, 2008; Zhang et al., 2020). Additionally, rather than functioning independently, LBD proteins may act through interaction networks involving ABA-related transcription factors or other stress-responsive regulators to coordinate the expression of downstream target genes. However, it should be noted that the present study is primarily based on differential gene-expression patterns observed in plants carrying the corresponding VIGS constructs, and the direct molecular mechanisms by which LBD genes contribute to ABA-mediated stress responses have yet to be experimentally verified. Future studies integrating target gene identification, protein interaction assays, and transcriptomic approaches will be necessary to elucidate the molecular regulatory mechanisms through which *LBD* genes mediate stress adaptation.

Low-temperature stress represents a widespread abiotic constraint that limits plant growth and productivity. It can trigger bursts of reactive oxygen species (ROS), causing oxidative damage, impairing vegetative growth and reproductive development, and substantially reducing crop yield and quality (Soualiou et al., 2022). Although studies on *LBD* genes in cold responses remain limited, evidence from rice (Sun et al., 2025), grapevine (Chen et al., 2024), and banana (Ba et al., 2016) has demonstrated that *LBD* members participate in cold responses and modulate cold tolerance. In this study, tobacco and eggplant seedlings carrying the respective *NtLBD4*/*NtLBD31* or *SmLBD19*/*SmLBD48* targeting VIGS constructs exhibited less severe visible injury under low-temperature treatment than the corresponding controls. These observations suggest that VIGS targeting these genes was associated with enhanced tolerance to low-temperature stress and that the corresponding *LBD* genes may be involved in the negative regulation of cold-stress responses. Consistently, the expression levels of several stress-related marker genes, including *NtPOD*, *NtSOD*, *SmAPX2*, *SmPAL5*, and *SmTAS14*, were lower in the corresponding VIGS plants than in the corresponding controls. This pattern suggests that the stronger induction of these marker genes in control plants may be associated with more severe cold-induced stress or cellular damage, whereas the weaker induction observed in the VIGS plants may reflect a reduced stress burden and consequently a lower requirement for the activation of stress-responsive transcriptional pathways. However, it should be noted that this conclusion is currently based mainly on visible phenotypes and stress-related gene expression patterns. As quantitative physiological indicators such as survival rate, electrolyte leakage, and proline content were not determined in this study, the contribution of these *LBD* genes to cold tolerance still requires further validation. Future studies integrating physiological measurements with molecular and biochemical analyses will be necessary to further elucidate the roles of these genes in cold-stress adaptation.

Bacterial wilt is a common disease of *Solanaceae* crops caused by the Gram-negative bacterium *Ras* (Yao and Allen, 2006). Members of the *LBD* transcription-factor family have been increasingly recognized as important regulators of plant immunity. For example, in *Arabidopsis thaliana*, *AtLBD20* is induced in roots upon *Fusarium oxysporum* infection and functions as a defense-related regulator modulated by JA and COI1, while also negatively influencing JA signaling (Thatcher et al., 2012). In cotton, *GhLBD41* enhances resistance to *Verticillium dahliae* by directly promoting JA biosynthesis (Hu et al., 2024), whereas in tomato, *SlLBD40*/*42* appear to antagonize host resistance (Zhang et al., 2025). These examples illustrate the diverse and sometimes opposing roles that LBD proteins can play in pathogen defense. Our results extend this functional landscape to two economically important *Solanaceae* species. VIGS targeting *NtLBD4/NtLBD31* in tobacco and *SmLBD19/SmLBD48* in eggplant was associated with markedly different responses to *Ras* challenge, with increased susceptibility observed in tobacco and enhanced resistance detected in eggplant. These contrasting phenotypic trends suggest that members of the *LBD* gene family do not play a conserved and uniform role in bacterial wilt responses, but may instead function in a species-dependent manner. In tobacco, *NtLBD4* and *NtLBD31* may be involved in the positive regulation of defense responses against bacterial wilt, whereas *SmLBD19* and *SmLBD48* may participate in the negative regulation of these responses in eggplant. Such opposing regulatory effects may reflect substantial divergence in the immune regulatory networks in which these LBD proteins function, and may be associated with differences in their downstream target genes and interacting partners. In addition, transcriptional changes among phylogenetically related *LBD* members in the corresponding VIGS plants suggest the possible presence of functional redundancy or buffering mechanisms within the *LBD* family. Such compensatory changes may partially mask or modify the phenotypic responses associated with VIGS targeting individual genes, thereby further increasing the complexity of the observed species-specific responses. Nevertheless, the present study mainly reveals an association between VIGS targeting the selected *LBD* genes and altered bacterial wilt phenotypes, and the molecular basis underlying these species-specific effects remains to be further elucidated.

## Conclusion

This study provides a comprehensive genomic characterization and preliminary functional assessment of the *LBD* transcription factor family in tobacco and eggplant. We identified 106 *NtLBD* and 50 *SmLBD* genes and classified them into two major groups, Classes I and II. Through the analysis of gene duplication events and *cis*-regulatory elements, we characterized the evolutionary expansion of the *LBD* families and identified their potential associations with hormone signaling, stress responses, and plant development. VIGS based analyses showed that targeting *NtLBD4*/*NtLBD31* in tobacco and *SmLBD19*/*SmLBD48* in eggplant was associated with distinct changes in ABA responsive gene expression, low-temperature responses, and resistance to bacterial wilt (**Figure 10**). Notably, plants carrying the corresponding VIGS constructs exhibited reduced visible injury under low-temperature treatment in both species, whereas contrasting responses to bacterial wilt were observed in tobacco and eggplant. These phenotypic changes were accompanied by transcriptional alterations in phylogenetically related *LBD* members, suggesting possible functional redundancy or compensatory regulation within the *LBD* family. Collectively, these findings provide preliminary evidence that the selected *LBD* genes and their associated regulatory networks participate in stress responses in *Solanaceous* plants. By integrating comparative genome-wide analysis with VIGS based functional evaluation in two *Solanaceae* species, this study extends *LBD* research beyond gene family characterization and expression profiling and provides preliminary experimental evidence for the involvement of selected *LBD* members in abiotic and biotic stress responses. However, because potential co-silencing of closely related homologs cannot be excluded, the individual functions of these genes require further validation using homolog-specific expression analyses, independent silencing fragments, genetic complementation, or stable gene-editing materials. Overall, our findings broaden the current understanding of stress-associated *LBD* regulation in *Solanaceae* and highlight several candidate genes for further functional validation and potential application in stress resilience.

**Figure 10:**
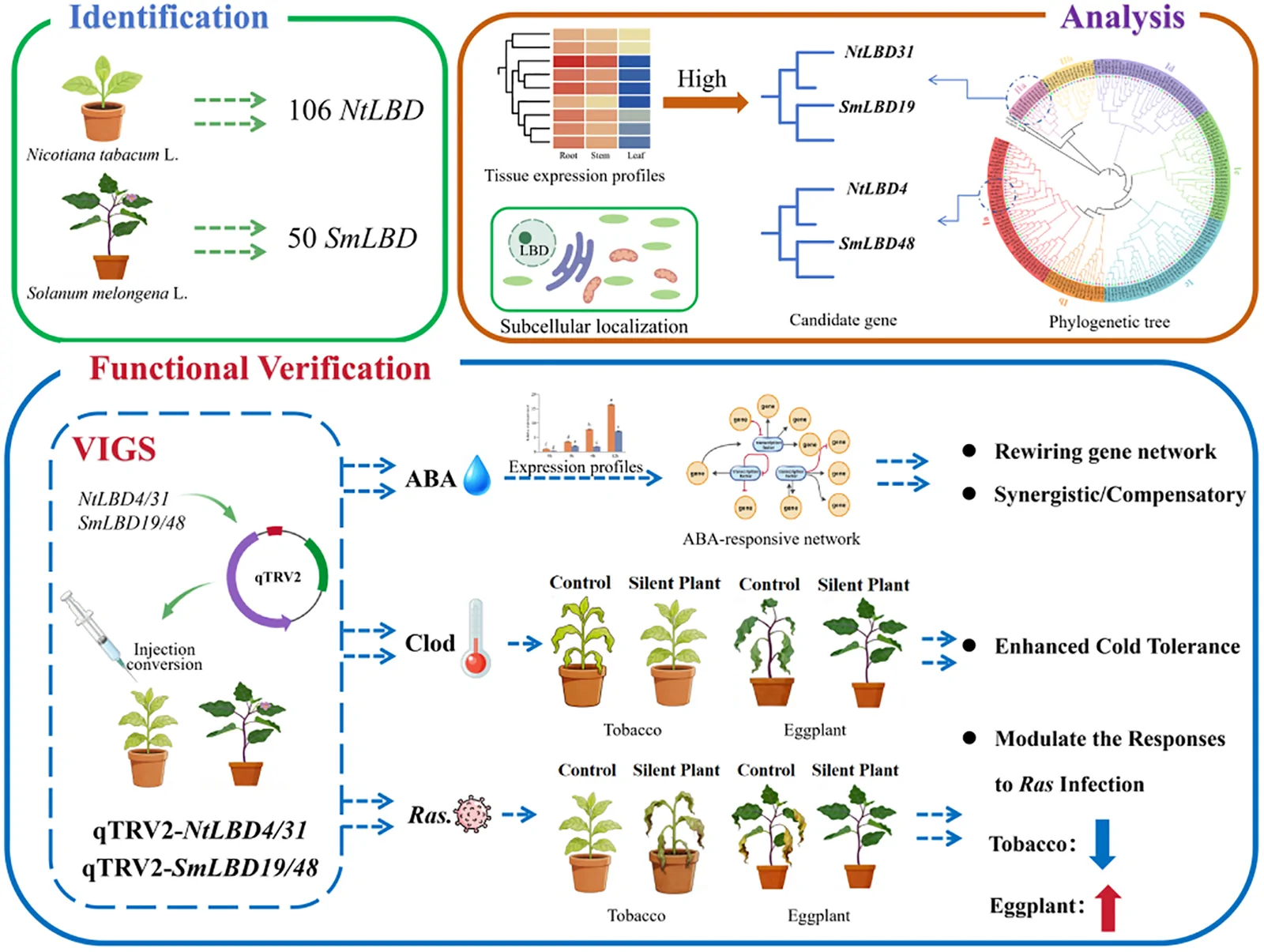
Graphical summary of *LBD* gene characterization and VIGS based functional analysis in tobacco and eggplant. A total of 106 *NtLBD* and 50 *SmLBD* genes were identified and classified into Classes I and II. VIGS analysis of *NtLBD4*/*NtLBD31* and *SmLBD19*/*SmLBD48* indicated their potential involvement in ABA, low-temperature, and bacterial wilt responses.

## Data Availability

The original contributions presented in the study are included in the article/**Supplementary Material**. Further inquiries can be directed to the corresponding authors.
