# Supplementary material for: Genome-wide identification and functional characterization of LBD transcription factors uncover their stress adaptation mechanisms in tobacco and eggplant
Source: Front Plant Sci. 2026 Sep 18;17:1958148. doi: 10.3389/fpls.2026.1958148 (PMC13631828; doi:10.3389/fpls.2026.1958148)
Supplement: Supplementary file 1 [file DataSheet1.docx]

**Supplementary Fig. 1.** *Cis*-element analysis of promoter regions of tobacco *LBD* family genes. (A) The types of various cis-elements in the promoters of *NtLBD* genes. Different colors represent the different types of *cis*-elements. (B) The relative proportions of different kinds of *cis*-elements in the promoters of *NtLBD* genes are displayed by pie chart. Annotations of *cis*-elements were listed in Additional file: (Supplementary Table S6).

**Supplementary Fig. 2.** *Cis*-element analysis of promoter regions of eggplant *LBD* family genes. (A) The types of various cis-elements in the promoters of *SmLBD* genes. Different colors represent the different types of *cis*-elements. (B) The relative proportions of different kinds of *cis*-elements in the promoters of *SmLBD* genes are displayed by pie chart. Annotations of *cis*-elements were listed in Additional file: (Supplementary Table S7).

**Supplementary Fig. 3.** Tissue-specific expression profiles of *LBD* genes. (A) 40 *NtLBD* genes of tobacco. (B) 24 *SmLBD* genes of eggplant. Expression levels for *LBD* genes were transformed by log10 ^(PFKM+1)^.

**Supplementary Fig. 4.** Validation of the pTRV2-based VIGS system. (A) Representative images of leaves from wild-type (WT) plants and pTRV2-infected plants carrying the GFP sequence, observed under UV illumination (365 nm). Green fluorescence in infected leaves indicates viral expression. The first two panels show leaves of tobacco plants, while the remaining ones are from eggplant plants. The green fluorescent foci highlighted by red boxes represent detectable GFP fluorescence signals. (B) Relative expression levels of target genes in VIGS plants at 10-15 days post-infiltration, as determined by qRT-PCR. Data are presented as mean ± SD (n = 3). Differences among groups were analyzed by one-way ANOVA. Asterisks indicate significant differences (*p* < 0.05, *p* < 0.01, *p* < 0.001, *p* < 0.0001).

**Supplementary Figure 5.** Quantification of *Ras* populations in rhizosphere soil after inoculation. (A) Representative plate from the control group irrigated with sterile water. (B) Representative plate from the *Ras* inoculated group. *Ras* colonies were identified by their characteristic morphology, with a pink center surrounded by a white margin. *Ras* population densities in the rhizosphere soil were determined using the serial-dilution plating method and expressed as CFU g⁻¹ dry soil.
